# Association between sex steroid hormones and subsequent hyperglycemia during pregnancy

**DOI:** 10.3389/fendo.2023.1213402

**Published:** 2023-09-08

**Authors:** Ying Meng, Loralei L. Thornburg, Kathleen M. Hoeger, Zorimar Rivera- Núñez, Amber Kautz, Adam T. Evans, Christina Wang, Richard K. Miller, Susan W. Groth, Thomas G. O’Connor, Emily S. Barrett

**Affiliations:** ^1^ School of Nursing, University of Rochester, Rochester, NY, United States; ^2^ Obstetrics and Gynecology, University of Rochester Medical Center, Rochester, NY, United States; ^3^ Biostatistics and Epidemiology, Rutgers School of Public Health, Piscataway, NJ, United States; ^4^ Environmental and Occupational Health Sciences Institute, Rutgers University, Piscataway, NJ, United States; ^5^ Public Health Sciences, University of Rochester Medical Center, Rochester, NY, United States; ^6^ Division of Endocrinology, Department of Medicine and Clinical and Translational Science Institue, The Lundquist Institute at Harbor-University of California, Los Angeles (UCLA) Medical Center, Torrance, CA, United States; ^7^ Department of Psychiatry, University of Rochester, Rochester, NY, United States; ^8^ Department of Neuroscience, University of Rochester Medical Center, Rochester, NY, United States; ^9^ Wynne Center for Family Research, University of Rochester Medical Center, Rochester, NY, United States

**Keywords:** sex steroid hormone, hyperglycemia, gestational diabetes, testosterone, estrogen

## Abstract

**Objective:**

Sex steroid hormones may play a role in insulin resistance and glucose dysregulation. However, evidence regarding associations between early-pregnancy sex steroid hormones and hyperglycemia during pregnancy is limited. The primary objective of this study was to assess the relationships between first trimester sex steroid hormones and the subsequent development of hyperglycemia during pregnancy; with secondary evaluation of sex steroid hormones levels in mid-late pregnancy, concurrent with and subsequent to diagnosis of gestational diabetes.

**Methods:**

Retrospective analysis of a prospective pregnancy cohort study was conducted. Medically low-risk participants with no known major endocrine disorders were recruited in the first trimester of pregnancy (n=319). Sex steroid hormones in each trimester, including total testosterone, free testosterone, estrone, estradiol, and estriol, were assessed using high-performance liquid chromatography and tandem mass spectrometry. Glucose levels of the 1-hour oral glucose tolerance test and gestational diabetes diagnosis were abstracted from medical records. Multivariable linear regression models were fitted to assess the associations of individual first trimester sex steroids and glucose levels.

**Results:**

In adjusted models, first trimester total testosterone (β=5.24, 95% CI: 0.01, 10.46, p=0.05) and free testosterone (β=5.98, 95% CI: 0.97, 10.98, p=0.02) were positively associated with subsequent glucose concentrations and gestational diabetes diagnosis (total testosterone: OR=3.63, 95% CI: 1.50, 8.78; free testosterone: OR=3.69; 95% CI: 1.56, 8.73). First trimester estrone was also positively associated with gestational diabetes (OR=3.66, 95% CI: 1.56, 8.55). In mid-late pregnancy, pregnant people with gestational diabetes had lower total testosterone levels (β=-0.19, 95% CI: -0.36, -0.02) after adjustment for first trimester total testosterone.

**Conclusion:**

Early-pregnancy sex steroid hormones, including total testosterone, free testosterone, and estrone, were positively associated with glucose levels and gestational diabetes in mid-late pregnancy. These hormones may serve as early predictors of gestational diabetes in combination with other risk factors.

## Introduction

Hyperglycemia, mainly caused by gestational diabetes mellitus (GDM), is a common metabolic complication during pregnancy (1, 2). GDM is associated with an increased risk of pregnancy related and neonatal outcomes, such as cesarean delivery, macrosomia, and neonatal hypoglycemia (1, 2). Furthermore, in the longer term, people diagnosed with GDM have a higher risk of progression to type 2 diabetes (T2DM), with around 19% of people with GDM develop T2DM after 5 years or more from delivery (3, 4). Children born to people with GDM have an increased risk of obesity, metabolic diseases and neurodevelopmental disorders (5, 6). Genetic predisposition, age, race/ethnicity, and obesity have been identified as risk factors for GDM (1, 7–9). Yet, the pathogenesis of GDM still is poorly understood.

GDM and T2DM are both characterized by insulin resistance (1, 7). Evidence suggests that endogenous sex steroid hormones (SSH), such as testosterone and estradiol, play important roles in glucose intolerance, insulin resistance and the development of T2DM in non-pregnant people (10–13). Additionally, people with hyperandrogenic conditions, such as polycystic ovary syndrome (PCOS) and congenital adrenal hyperplasia, have a higher risk of insulin resistance and T2DM (14–16). Lowering androgen production in PCOS patients leads improved insulin sensitivity and reduces fasting insulin levels (17, 18). Postmenopausal hormone therapy with estrogen/progestin reduces the incidence of diabetes (19, 20). Therefore, through their impacts on insulin and glucose metabolism, endogenous SSH may be involved in the pathogenesis of T2DM.

Likewise, SSH may play a role in the development of GDM. Nevertheless, pregnancy is a unique period given the rapid hormonal changes and the substantially increased estrogen concentrations (21), which may affect the relationship between SSH and glucose regulation. Evidence from people with PCOS substantiates the link between SSH and the risk of GDM during pregnancy (22, 23). However, to date, very few prospective studies have assessed the involvement of SSH, including testosterone and estriol (E3), in the development of GDM in people without PCOS (24–27). Yet, these previous studies have only examined total testosterone (TT) rather than free testosterone (fT) which represents the biologically active fraction of testosterone. Also, these studies did not concurrently examine multiple estrogens as well as testosterone despite their interrelatedness.

Additionally, the association between SSH and GDM may be bidirectional, operating through adipose tissue and insulin regulation (28, 29). Insulin induces androgen biosynthesis in cultured human ovarian theca and stromal cells (30), which suggests that GDM could in turn alter androgen production. Several small case-control studies have assessed differences in SSH in late pregnancy, subsequent to GDM diagnosis, with inconsistent findings (31–33). Moreover, the previous studies did not consider the potential confounding effect of early-pregnancy SSH on the relationship between GDM and SSH in late pregnancy.

Here, we leverage data and biospecimens from a pregnancy cohort that was medically not greater than normal risk at baseline with no known preexisting hormonal conditions to assess testosterone (fT and TT) and estrogens (estrone, estradiol, E3) in early pregnancy in relation to glucose concentrations and GDM diagnosis assessed in mid-late pregnancy. Secondarily, we evaluated associations between GDM diagnosis and the same set of SSH assessed later in pregnancy with and without adjusting for early-pregnancy SSH levels.

## Materials and methods

### Study overview

**Figure 1:**
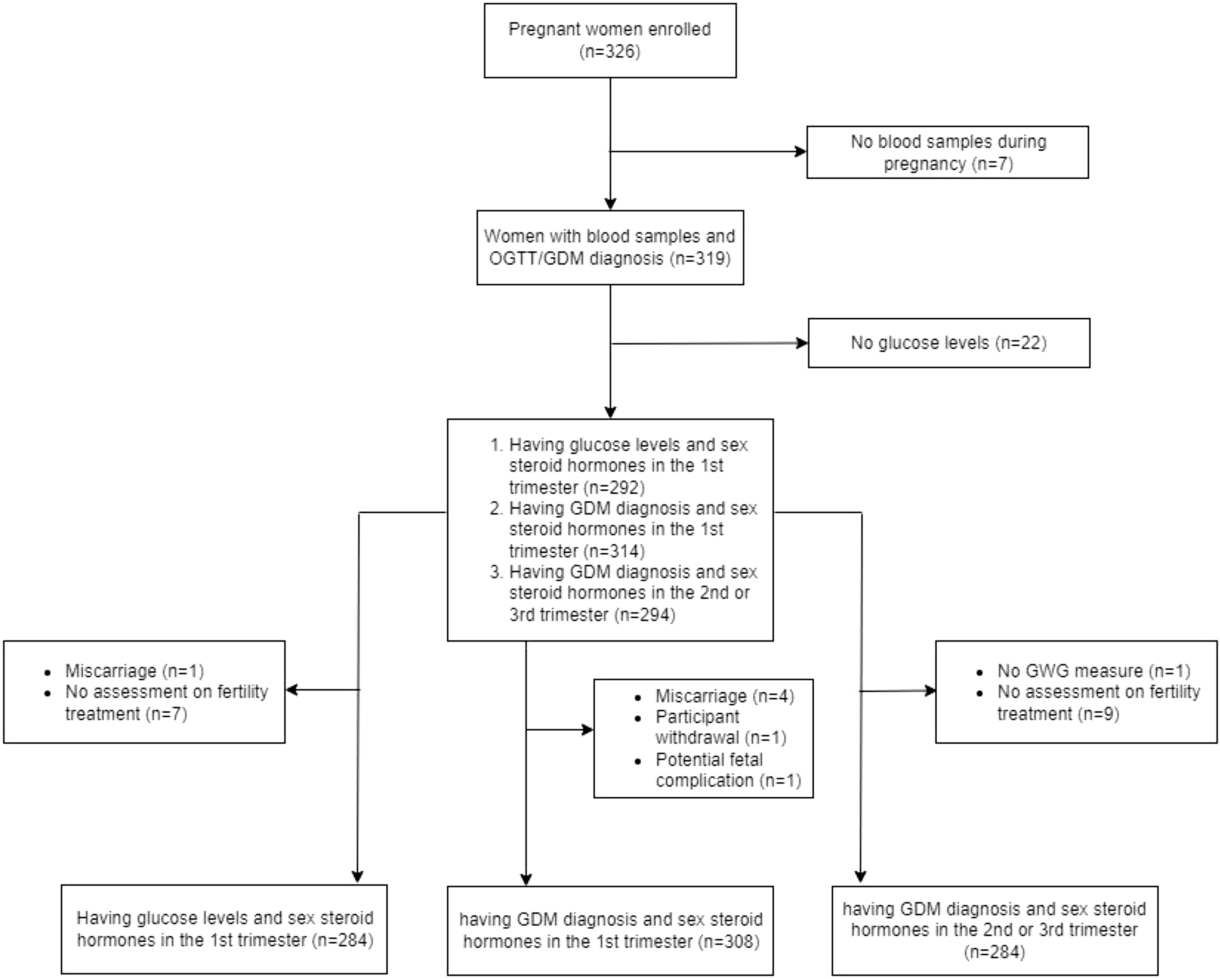
Flow chart displaying inclusion and exclusion of this study. GDM, gestational diabetes.

The current study is a retrospective analysis of a prospective pregnancy cohort, the Understanding Pregnancy Signals and Infant Development (UPSIDE) study that is a part of the Environmental Influences on Child Health Outcomes (ECHO) program (34). From 2015 to 2019, the UPSIDE study recruited pregnant people (n=326) in their first trimester receiving prenatal care through the University of Rochester Medical Center affiliated obstetric clinics (35). Briefly, the inclusion criteria for the UPSIDE study were (1) <14 weeks of gestation, (2) age 18 or older, (3) a singleton pregnancy, (4) able to communicate in English, (5) no known substance abuse problems or history of psychotic illness, and (6) no greater than normal medical risk. Additionally, women with diagnosed PCOS and T2DM were excluded from the cohort. The study was approved by the institutional review boards at the University of Rochester and Rutgers University. All participants provided written informed consent prior to participation. The current analysis included participants with SSH measured during pregnancy and a 1-hour oral glucose tolerance test (OGTT) or GDM diagnosis (n=319; **Figure 1**).

### Sex steroid hormone assays

Blood samples were collected in each trimester (1^st^ trimester: 12.2 ± 1.3 weeks; 2^nd^ trimester: 21.2 ± 1.8 weeks; 3^rd^ trimester: 31.4 ± 2 weeks) and after processing, serum was stored in a -80°C freezer until overnight shipment to the Endocrine and Metabolic Research Laboratory at Harbor-UCLA Medical Center. SSH, including TT, fT, estrone(E1), estradiol(E2), and E3, were quantified using validated liquid chromatography with tandem mass spectrometry (LC-MS/MS) methods (36). Briefly, LC–MS/MS was used to assess testosterone concentrations using a Shimadzu HPLC system (Columbia, MD) and an Applied Biosystems API5500 LC–MS/MS (Foster City, CA) equipped with a Turbo-Ion-Spray source that used positive mode. Quality control was performed on each assay run using spiked samples. The limit of quantification (LOQ) for TT was 2 ng/dL. Equilibrium dialysis using labeled testosterone was used to measure fT% which is used to calculate fT levels (fT=TT x fT%). fT% was not detected in one sample collected in the 1^st^ trimester. The Shimadzu HPLC system (Columbia, MD) and a triple quadrupole mass spectrometer (API5000 LC–MS/MS, Foster City, CA) were used to measure estrogen concentrations. The LOQ was 2 pg/mL for E1 and E2, and 50 pg/mL for E3. E3 was not detected in 32 samples collected in the 1^st^ trimester $\text{LOQ}/\sqrt{2}$ was used to replace missing E3 values (n=32) and E3 values less than $\text{LOQ}/\sqrt{2}$ . We additionally calculated the ratio of TT to E2 as a measure of hormone balance.

### Glucose measures

As part of routine obstetric care, participants were screened for GDM with 1-hour 50g OGTT at an average gestational age of 27.7 weeks ( ± 2.9 weeks). Participants with a 1-hour OGTT value of more than 135 mg/dL underwent a further diagnostic test with 3-hour 100g OGTT. Per clinical protocols, GDM was diagnosed according to the National Diabetes Data Group (NDDG) criteria: if the 3-hour OGTT values met more than two of the following values: fasting, 105 mg/dL; 1 hour, 190 mg/dL; 2 hours, 165 mg/dL; and 3 hours, 145 mg/dL. Several participants (n=5) were diagnosed with GDM without completing the 3-hour OGTT by either (1) 1-hour OGTT >200 mg/dL, (2) fasting glucose levels >125 mg/dL, or (3) by paneled blood glucose levels due to inability to complete 3-hour OGTT because of intolerance or history of gastric bypass surgery. OGTT values and GDM diagnosis were abstracted from electronic medical records by trained study staff.

For the purpose of this study, we additionally considered the Carpenter-Coustan (CC) criteria which may identify more GDM cases (37, 38). CC criteria use lower threshold values: if the 3-hour OGTT values met more than two of the following values: fasting, 95 mg/dL; 1 hour, 180 mg/dL; 2 hours, 155 mg/dL; and 3 hours, 140 mg/dL. Six additional participants were classified as having GDM based on the CC criteria.

### Body weight measures and other covariates

Adipose tissue may be involved in the metabolism of SSH (39–41) and glucose dysregulation (28, 42, 43). We, therefore, included early-pregnancy body mass index (BMI) as a key confounder in the analyses. Early-pregnancy BMI, used as a proxy for pre-pregnancy BMI, was calculated based on weight and height abstracted from medical records from the first clinical visit prior to 14 weeks gestation and the formula $BMI=\frac{Weight(kg)}{Height{\left(m\right)}^{2}}$ (44).

SSH have been linked to adiposity (45) and early excess gestational weight gain (GWG) has been associated with GDM (46). Therefore, early GWG through the end of 2^nd^ trimester was explored as a potential mediator between the associations of first trimester SSH and GDM. GWG through the end of 2^nd^ trimester was calculated as weight at the end of the 2^nd^ trimester minus early-pregnancy weight. Additionally, GWG through the end of 2^nd^ trimester and total GWG until delivery were included as confounders in our secondary analyses of associations between GDM diagnosis and SSH assessed in mid-late pregnancy.

Age, race/ethnicity, parity, gestational age at the time of blood sample collection, fertility treatment, and infant sex, have been associated with SSH levels during pregnancy and in some cases, GDM as well, and were thus included as covariates (8, 47). Race/ethnicity was categorized as non-Hispanic White, non-Hispanic Black, Hispanic, and others. Parity was characterized as nulliparous and parous. Gestational dating was based on crown-rump length at the earliest available ultrasound and last menstrual period was used when an early ultrasound was not available (7%). Fertility treatment (any/none) was classified based on participant self-report. Although participants diagnosed with PCOS were excluded from the UPSIDE study, to address the possibility of undiagnosed cases, participants were evaluated with several questions to address relevant symptoms, including regularity of periods, hirsutism and acne (see **Supplementary Materials**) (48). Participants (n=13) categorized as potentially undiagnosed PCOS cases and were excluded in the sensitivity analyses. Additionally, four participants reported having a history of GDM in previous pregnancies and were excluded in the sensitivity analyses.

### Statistical analysis

Descriptive statistics were calculated for all variables of interest. SSH were not normally distributed and were thus log-transformed. Early-pregnancy BMI was right skewed and was inverse-transformed. In the primary analyses, a multivariable linear regression model was fitted to assess the association of each first trimester SSH and glucose levels (continuous variable) based on routine 1-hour OGTT. A logistic regression model was fitted to assess the association of each first trimester SSH and GDM diagnosis. Age, race/ethnicity, parity, gestational age at the time of blood sample collection, fertility treatment, early-pregnancy BMI, and infant sex were included as covariates. Fertility treatment was not included in logistic regression models as no positive GDM cases were diagnosed in people reporting fertility treatment for the current pregnancy. GWG through the end of 2^nd^ trimester was further assessed as a potential mediator of the associations between first trimester SSH and GDM diagnosis (**Supplementary Figure 1**) with bootstrap to estimate bias-corrected confidence intervals (CI). In secondary analyses, linear mixed effects models were fitted to assess the associations of GDM diagnosis and individual SSH in the 2^nd^ and 3^rd^ trimesters. Age, race/ethnicity, parity, gestational age at the time of blood sample collection, fertility treatment, infant sex, early-pregnancy BMI and GWG were included as covariates. First trimester SSH was additionally included as a key confounder. All analyses were conducted using STATA 17.0 (College Station, TX: StataCorp LLC).

## Results

### Characteristics of the study cohort

The majority of participants (n=319) were non-Hispanic White (55.5%), had at least one prior birth (65.2%), had a college education or more (62.0%), and were overweight or obese in early pregnancy (57.6%). Twenty-two participants (6.9%) were classified as having GDM in this study. The characteristics of the participants grouped by GDM diagnosis are described in **Table 1**. Participants with GDM were slightly older that those without GDM (30.95 ± 0.71 vs 28.66 ± 0.27 years, p=0.005). SSH varied significantly across trimesters except for fT (**Supplementary Table 1**). Trend tests indicated that E1, E2, and E3 levels increased and TT/E2 ratios decreased across pregnancy (p<0.001). The correlations among first trimester SSH were weak to moderate (r=0.17-0.35) except for the high correlations between TT and fT (r=0.91) and between E1 and E2 (r=0.81). The correlation between TT and E3 was not significant (**Supplementary Table 2**).

**Table 1:** Characteristics of UPSIDE Participants (n=319).

Variable^a^	All Participants (n=319)^b^	Participants with GDM (n=22)	Participants without GDM (n=297)^c^
Age (years)	28.82 **±** 4.68	30.95 ± 3.33	28.66 ± 4.73
Race/Ethnicity			
White, Non-Hispanic	177 (55.5%)	14 (63.6%)	163 (54.9%)
Black, Non-Hispanic	82 (25.7%)	3 (13.6%)	79 (26.6%)
Hispanic	34 (10.7%)	3 (13.6%)	31 (10.4%)
Others	26 (8.2%)	2 (9.1%)	24 (8.1%)
Nulliparous	110 (34.8%)	10 (45.5%)	100 (34%)
Education			
High school or less	120 (38.0%)	8 (36.4%)	112 (38.1%)
Fetal sex_male	158 (50.5%)	10 (45.5%)	148 (50.9%)
Early-pregnancy BMI (kg/m^2^)	28.27 **±** 7.04	30.65 ± 8.34	28.09 ± 6.92
Glucose levels^d^ (mg/dL)	113.66 ± 26.32	157.82 ± 16.58	110.13 ± 23.63

^a^Continuous variables are summarized using mean and standard deviation; Categorical variables are summarized using count and percentage. ^b^Sample size for parity, education and early-pregnancy BMI is 316; sample size for infant sex is 313; sample size for glucose levels is 297. ^c^Sample size for parity, education and early-pregnancy body mass index (BMI) is 294; sample size for infant sex is 291; sample size for glucose level is 275. ^d^Glucose levels were derived from 1-hour glucose tolerance test results.

### Associations of first trimester sex steroid hormones with mid-late pregnancy glucose levels and GDM diagnosis

**Figure 2:**
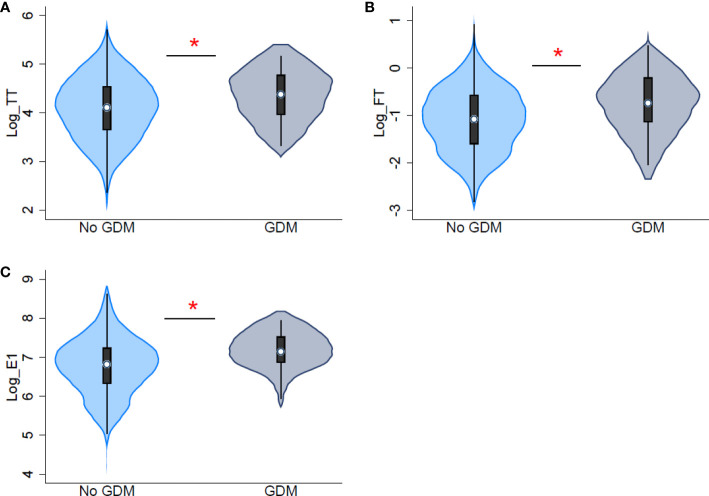
Distribution of first trimester log-transformed sex steroid hormones by gestational diabetes diagnosis. **(A)** distribution of total testosterone (TT) **(B)** distribution of free testosterone (FT) **(C)** distribution of estrone (E1). * indicates significant differences between participants with and without gestational diabetes (GDM) diagnosis.

In the primary multivariable regression models, first trimester TT and fT were positively associated with glucose levels measured in mid-late pregnancy after adjusting for maternal age, race/ethnicity, parity, gestational age of blood draw, early-pregnancy BMI, fertility treatment, and infant sex (**Table 2**). One natural-log unit increases in TT and fT were associated with 5.24 mg/dL (TT: 95% CI: 0.01, 10.46, p=0.05) and 5.98 mg/dL (fT: 95% CI: 0.97, 10.98, p=0.02) higher glucose levels, respectively. Associations between first trimester estrogens and glucose levels were also positive but slightly weaker. Higher first trimester TT and fT was also associated with increased odds of GDM diagnosis (TT: OR=3.63, 95% CI: 1.50, 8.78, p=0.004, **Figure 2A**; fT: OR=3.69, 95% CI: 1.56, 8.73, p=0.003, **Figure 2B**). Higher first trimester E1 (OR=3.66, 95% CI: 1.56, 8.55, p=0.003, **Figure 2C**) and E2 (OR=2.92, 95% CI: 1.00, 8.55, p=0.05), but not E3, were also associated with higher odds of GDM diagnosis. Exclusion of potentially undiagnosed PCOS cases in sensitivity analyses slightly strengthened associations between testosterone and E1 concentrations and glucose levels/GDM diagnosis (**Supplementary Table 3**). Exclusion of participants with a history of GDM during previous pregnancies had similar results on the associations of fT and E1 with glucose levels and GDM diagnosis (**Supplementary Table 4**). Associations of TT, fT, and E1 with clinical GDM diagnosis (solely by clinical criteria, not CC criteria) remained significant (**Supplementary Table 5**). Given the relatively weak correlations between testosterone and estrogens, we explored models including both fT and E1 simultaneously, fT and E1 were still associated with higher odds of GDM diagnosis (fT: OR=3.33, 95% CI: 1.35, 8.23, p=0.009; E1: OR=3.32, 95% CI: 1.38, 8.03 p=0.008); associations with glucose levels were positive but attenuated compared to models assessing the hormones individually (fT: β =5.12, 95% CI: -0.02, 10.26, p=0.05; E1: β =3.31, 95% CI: -1.35, 7.97, p=0.16).

**Table 2:** Associations of Log-transformed First Trimester Sex Steroid Hormones with Glucose Levels and Gestational Diabetes Diagnosis in Mid-late Pregnancy.

Sex Steroid Hormones	Glucose Levels (mg/dL) (n=284)			GDM Diagnosis (n=308)		
	Coefficient	95% CI	*P*	OR	95% CI	*P*
TT (ng/dL)	5.24	0.01, 10.46	0.05	3.63	1.50, 8.78	0.004
fT (ng/dL)	5.98	0.97, 10.98	0.02	3.69	1.56, 8.73	0.003
E1 (pg/mL)	4.39	-0.15, 8.94	0.06	3.66	1.56, 8.55	0.003
E2 (pg/mL)	5.65	-1.02, 12.31	0.10	2.92	1.00, 8.55	0.05
E3 (pg/mL)	2.99	-0.17, 6.14	0.06	1.06	0.66, 1.71	0.82
TT/E2	1.68	-3.46, 6.83	0.52	1.62	0.77, 3.44	0.21

Maternal age, race/ethnicity, parity, gestational age of blood draw, early-pregnancy BMI, and infant sex were adjusted in all models. Fertility treatment was adjusted in the models with glucose levels as the outcome. All sex steroids were log-transformed. GDM, gestational diabetes; TT, total testosterone; fT, free testosterone; E1, estrone; E2, estradiol; E3, estriol.

### Evaluation of confounding and mediation by adiposity

Early-pregnancy BMI was a key confounding variable in the associations between sex steroids and glucose levels. Early-pregnancy BMI was positively associated with glucose levels (β=0.65, 95% CI: 0.21, 1.08, p=0.004) and first trimester fT and TT/E2 ratio, but was negatively associated with E1 and E2 (**Supplementary Table 6**). Regression models including early-pregnancy BMI as a covariate (**Table 2**) showed similar but slightly weakened significant positive associations between testosterone and glucose levels/GDM compared to regression models excluding early-pregnancy BMI (**Supplementary Table 7**). The relationships among early-pregnancy fT, early-pregnancy BMI and GDM are also illustrated in **Supplementary Figure 2**. The association between E1 and GDM was attenuated (OR=2.95, 95% CI: 1.31, 6.64, p=0.01) by excluding early-pregnancy BMI in the models (**Supplementary Table 7**).

GWG might mediate the effect of sex steroids on glucose levels. But GWG through the end of the 2^nd^ trimester was not significantly associated with GDM diagnosis (OR=0.96, p=0.15) and only showed a borderline association with first trimester TT (β=1.61, 95% CI: -0.08, 3.30, p=0.06). The mediation effect of GWG on the relationship between TT and GDM was not significant (indirect effect: β=-0.05, 95% CI_bootstrap_: -0.22, 0.03).

### Associations of GDM diagnosis with sex steroid hormones in the 2^nd^ and 3^rd^ trimesters

GDM diagnosis was positively associated with E1 levels (β=0.29, 95% CI: 0.02, 0.56, p=0.03) in the 2^nd^ and 3^rd^ trimesters (**Supplementary Table 8**). Further adjusting for E1 levels in the 1^st^ trimester, the association between GDM and E1 levels in the 2^nd^ and 3^rd^ trimesters was not significant (β=0.01, 95% CI: -0.18, 0.19, p=0.95). However, GDM diagnosis was inversely associated with TT in the 2^nd^ and 3^rd^ trimesters (β=-0.19, 95% CI: -0.36, -0.02, p=0.03), after adjustment for first trimester TT. But no associations between GDM and fT in the 2^nd^ and 3^rd^ trimesters were observed.

## Discussion

In this prospective pregnancy cohort including pregnant people who were medically not greater than normal risk at enrollment, first trimester TT, fT, and E1 were positively associated with glucose levels and GDM diagnosis in mid-late pregnancy, with similar trends observed for E2. fT and E1 were independently associated with increased odds of subsequent GDM diagnosis, when both were included in the same model. Results were robust to the exclusion of participants with potentially undiagnosed PCOS. GDM diagnosis was associated with lower TT but not fT levels in the 2^nd^ and 3^rd^ trimesters, when first trimester SSH was adjusted, respectively.

In females, androgens are mainly produced by the ovaries, adrenal glands, and adipose tissue (49). The placenta may also contribute to androgen synthesis during pregnancy (50). Prior studies that assessed associations between first trimester androgen levels and subsequent GDM diagnosis are limited. Two studies found a positive relationship between total testosterone levels in early pregnancy and GDM diagnosis in White pregnant people (25, 26), consistent with the results of this study. However, Gözükara, et al. (2015) and Mustaniemi, et al. (2023) measured TT levels using immunoassays and did not directly measure fT, the biologically active form of testosterone (25, 26). Improving upon the limitations of immunoassays, this study used LC-MS/MS, a gold standard method with greater sensitivity and specificity for steroid measurement (51). Similar to TT, first trimester fT showed positive and slightly stronger associations with glucose levels and GDM diagnosis.

Although evidence of the associations of first trimester TT and fT with GDM diagnosis is scarce, in prospective studies of non-pregnant people, TT and/or fT have been positively associated with development of T2DM in pre- and post-menopausal people (10, 12, 52, 53); but other studies have observed either no or attenuated associations after adjusting for adiposity (54–56). Generally, concentrations of TT and fT are higher in pregnant people compared to non-pregnant people (49), so to the extent that androgens play a causal role in glucose dysregulation, pregnancy may be a period of particular vulnerability.

We observed little evidence that adiposity was a confounder or mediator of the relationship between early-pregnancy testosterone and the development of GDM. Gözükara, et al. (2015) and Mustaniemi, et al. (2023) also identified that early pregnancy TT levels were higher among participants who subsequently developed GDM after adjusting for BMI (25, 26), which was consistent with our findings. However, evidence suggests that androgens exert direct and indirect effects on insulin sensitivity in adipose tissue and skeletal muscle (28, 42, 43). In female animal models, testosterone administration increased insulin resistance with or without western diet (57, 58). In subcutaneous adipocytes harvested from healthy non-pregnant people, testosterone treatment induced insulin resistance *in vitro* and inhibited insulin-stimulated glucose uptake (59). Administration of testosterone to oophorectomized female rodents impaired whole-body insulin-mediated glucose uptake potentially by lessening glycogen synthase expression and GLUT4 transporter expression in skeletal muscle (60–62). Also, anti-androgen treatments improved glucose tolerance in pregnant rat models (63). Given the findings in this study and the research in animal models and human adipocytes, it is postulated that androgens may contribute to the development of GDM by inducing insulin resistance not only in adipose tissue but also in other tissues, such as skeletal muscle.

In pregnant people, estrogens are mainly produced by the ovaries and placenta, with smaller contributions from other tissues, such as adipose tissue and adrenal glands (24). In this study, first trimester E1 levels were positively associated with subsequent GDM diagnosis. Although excessive testosterone could be converted into E1 in adipose tissue (43), in this study E1 was found to be a predictor of GDM independent of fT. We know of no other study that has addressed this association previously, but in a study of non-pregnant premenopausal people, estrone sulfate levels were positively correlated with postprandial glucose levels (56). In non-pregnant premenopausal people with PCOS, higher E1/E2 ratio was associated with increased fasting and postprandial glucose levels and insulin resistance (64). Therefore, E1 is potentially involved in glucose intolerance and GDM.

Research on the mechanisms linking estrogens to glucose regulation has primarily focused on E2 and evidence on E1 is sparse (65). In this study, while all estrogens showed positive associations with glucose levels and GDM, associations were strongest for E1. Borthwick et al. (2001) found that estrone sulfate could normalize hyperglycemia in obese-diabetic mice (both male and female) via the reduction of hepatic glucose-6-phosphatase (66). Although this finding conflicts with our results and findings in premenopausal women (56, 64), it is consistent with findings on E2, which may protect pancreatic β cell functions (67–69), reduce adipocyte hypertrophy and insulin resistance (68, 70), and improve hepatic glucose utilization (71). On the other hand, high concentrations of endogenous E2, particularly seen during pregnancy (21), may reduce insulin sensitivity (72) via decreased GLUT4 transporter expression in skeletal muscle (73) and interfere with insulin binding to insulin receptors (74). Therefore, the effect of endogenous estrogens on glucose regulation may vary in a non-linear manner and high concentrations of E1, similar to E2, potentially induce insulin resistance during pregnancy.

Because GDM may affect the production of SSH via insulin (30, 75), we further assessed the associations of GDM with SSH in mid-late pregnancy. When first-trimester SSH was not considered, the associations between GDM and SSH levels in mid-late pregnancy were consistent with the directions of associations between early-pregnancy SSH and GDM. These results were also similar to previous findings (31–33). When first-trimester estrogen was considered, the associations between GDM and estrogen were greatly attenuated, which indicates that the positive associations in mid-late pregnancy could be accounted by or driven by early-pregnancy estrogen levels. When first-trimester testosterone was considered, the directions of associations between GDM and testosterone were reversed, although the association between GDM and fT was not significant. These findings indicate that other factors changing during mid-late pregnancy, such as insulin levels which may be affected by GDM treatment, sex hormone binding globulin (SHBG) levels which is bound to fT to form TT, placental aromatase, and increasing gestational weight, may affect mid-late pregnancy testosterone levels and thus the relationship between GDM and mid-late pregnancy testosterone levels (30, 75–77).

A strength of this study is the measurement of SSH using the gold standard LC-MS/MS method, which is an advance over prior studies in this field. Furthermore, the prospective design of the study cohort established the temporal relationships between SSH in the 1^st^ trimester and glucose levels and GDM in mid-late pregnancy. In addition, repeat measures of SSH throughout pregnancy enabled us to assess hormone levels both prior to and after GDM diagnosis, while taking early-pregnancy SSH levels into consideration. Several limitations should be considered when interpreting the results of the current analyses. We did not assess insulin resistance or visceral adiposity in our cohort, which are potential key mechanisms linking SSH to GDM (1, 7, 24, 42). Further investigations of the relationship among SSH, adiposity, and insulin resistance during pregnancy are warranted. Also, future studies could assess the effect of insulin and SHGB levels during mid-late pregnancy on the relationship between GDM and mid-late pregnancy testosterone levels. Another limitation is that the limited GDM cases in this study could not provide reliable estimations of the cutoff values of first trimester TT, fT or E1 to predict GDM. Additionally, we did not assess SHBG, which was negatively associated with GDM in a recent meta-analysis (78). SHBG binds both testosterone and E2 during pregnancy (42, 79) and thus, low SHBG levels indicate high serum concentrations of fT and free E2. Therefore, the previous findings of the negative association between SHBG and GDM are consistent with the positive associations between fT and GDM found in this study (78). We assessed the potential undiagnosed PCOS cases by self report using a two-question response to oligomenorrhoea and hirsutism. This self-report approach has been found in longitudinal studies to be associated with clinical biomarkers and measures (48, 80), although additional assessments could confirm the diagnosis.

## Conclusion

In this prospective study of pregnant people, higher levels of first-trimester TT, fT and estrone were positively associated with glucose levels and GDM diagnosis in mid-late pregnancy. Our findings suggest that the early-pregnancy hormonal milieu may contribute to and/or predict gestational hyperglycemia. Studies such as the current study that identify early-pregnancy biomarkers may inform future targeted screening and interventions (lifestyle modifications, etc.) aimed at preventing GDM in pregnant people who are at risk.

## Data availability statement

The datasets presented in this study can be found in online repositories. The names of the repository/repositories and accession number(s) can be found below: https://dash.nichd.nih.gov/study/417122.

## Ethics statement

The studies involving humans were approved by The University of Rochester Research Subjects Review Board. The studies were conducted in accordance with the local legislation and institutional requirements. Written informed consent for participation in this study was provided by the participants’ legal guardians/next of kin.

## Author contributions

All authors contribute to the generation of hypotheses, statistical analyses, manuscript preparation and final approval of the manuscript. All authors contributed to the article and approved the submitted version.
