# Supplementary material for: Association between sex steroid hormones and subsequent hyperglycemia during pregnancy
Source: Front Endocrinol (Lausanne). 2023 Sep 8;14:1213402. doi: 10.3389/fendo.2023.1213402 (PMC10520461; doi:10.3389/fendo.2023.1213402)
Supplement: Supplementary file 1 [file DataSheet_1.docx]

Supplementary Table 1. Sex Steroid Hormone Concentrations in Pregnancy by Trimester in the UPSIDE Cohort.

| Sex Steroid Hormone | Trimester 1 (n=314) | Trimester 2 (n=287) | Trimester 3 (n=283) | *P^b^* |
| --- | --- | --- | --- | --- |
|  | Median (IQR)^a^ | | |  |
| Total testosterone (TT) (ng/dL) | 63.9 (56.8) | 62.9 (59.3) | 64.3 (63.6) | <0.001 |
| Free testosterone (fT) (ng/dL) | 0.35 (0.36) | 0.31 (0.31) | 0.29 (0.34) | 0.07 |
| Estrone (E1) (pg/mL) | 936.5 (860) | 3550 (2910) | 5910 (4560) | <0.001 |
| Estradiol (E2) (pg/mL) | 1625 (1180) | 5970 (3320) | 11100 (5420) | <0.001 |
| Estriol (E3) (pg/mL) | 211.5 (405) | 3130 (1480) | 6670 (3470) | <0.001 |
| TT/E2 ratio | 0.37 (0.35) | 0.1 (0.09) | 0.05 (0.06) | <0.001 |

Note. ^a^Present the median and interquartile range (IQR) because sex steroid hormone levels were skewed. ^b^Multivariate tests of means to assess the hypothesis that means of log transformed sex steroid hormones at different trimesters are equal.

Supplementary Table 2. Correlations of first trimester log-transformed sex steroid hormones (n=314).

|  | TT | fT | E1 | E2 |
| --- | --- | --- | --- | --- |
| fT | 0.91 (p<0.001) | - | - | - |
| E1 | 0.30 (p<0.001) | 0.17 (p=0.003) | - | - |
| E2 | 0.35 (p<0.001) | 0.22 (p<0.001) | 0.81 (p<0.001) | - |
| E3 | -0.06 (p=0.27) | -0.18 (p=0.002) | 0.19 (p<0.001) | 0.30 (p<0.001) |

Note. Correlation coefficient and p value are presented for each correlation analysis.

Supplementary Table 3. Sensitivity analysis of the associations of first trimester sex steroid hormones with glucose levels and GDM measured in mid-late pregnancy.

| Sex Hormones | Glucose levels (n=272) | | | GDM Diagnosis (n=295) | | |
| --- | --- | --- | --- | --- | --- | --- |
|  | Coefficient | 95% CI | *P* | OR | 95% CI | *P* |
| TT (ng/dL) | 5.88 | 0.45, 11.3 | 0.03 | 4.09 | 1.58, 10.56 | 0.004 |
| fT (ng/dL) | 6.65 | 1.47, 11.82 | 0.01 | 4.02 | 1.57, 10.29 | 0.004 |
| E1 (pg/mL) | 4.38 | -0.26, 9.02 | 0.06 | 3.77 | 1.55, 9.18 | 0.003 |
| E2 (pg/mL) | 5.24 | -1.56, 12.04 | 0.13 | 2.61 | 0.87, 7.82 | 0.09 |
| E3 (pg/mL) | 3.05 | -0.2, 6.29 | 0.07 | 1.07 | 0.65, 1.75 | 0.8 |
| TT/E2 | 2.44 | -2.94, 7.81 | 0.37 | 1.85 | 0.83, 4.12 | 0.13 |

Note. Sensitivity analysis excluded potential undiagnosed PCOS cases. Maternal age, race/ethnicity, parity, gestational age of blood draw, early-pregnancy BMI, and infant sex were adjusted in all models. Fertility treatment was adjusted in the models with glucose levels as the outcome. GDM: gestational diabetes; TT: total testosterone; fT: free testosterone; E1: estrone; E2: estradiol; E3: estriol.

Supplementary Table 4. The associations of first trimester sex steroid hormones with glucose levels and GDM measured in mid-late pregnancy excluding participants with history of GDM.

| Sex Hormones | Glucose levels (n=280) | | | GDM Diagnosis (n=304) | | |
| --- | --- | --- | --- | --- | --- | --- |
|  | Coefficient | 95% CI | *P* | OR | 95% CI | *P* |
| TT (ng/dL) | 4.44 | -0.7, 9.58 | 0.09 | 3.47 | 1.34, 8.97 | 0.01 |
| fT (ng/dL) | 5.3 | 0.38, 10.22 | 0.04 | 3.77 | 1.47, 9.64 | 0.006 |
| E1 (pg/mL) | 4.6 | 0.1, 9.1 | 0.05 | 4.23 | 1.62, 11.04 | 0.003 |
| E2 (pg/mL) | 5.13 | -1.43, 11.68 | 0.13 | 2.7 | 0.85, 8.59 | 0.09 |
| E3 (pg/mL) | 2.32 | -0.81, 5.45 | 0.15 | 0.85 | 0.51, 1.41 | 0.54 |
| TT/E2 | 1.22 | -3.82, 6.26 | 0.63 | 1.61 | 0.72, 3.6 | 0.25 |

Note. Sensitivity analysis excluded participants with history of GDM. Maternal age, race/ethnicity, parity, gestational age of blood draw, early-pregnancy BMI, and infant sex were adjusted in all models. Fertility treatment was adjusted in the models with glucose levels as the outcome. GDM: gestational diabetes; TT: total testosterone; fT: free testosterone; E1: estrone; E2: estradiol; E3: estriol.

Supplementary Table 5. Odds of clinical GDM diagnosis in relation to first trimester log-transformed sex steroid hormone concentrations.

| Sex Steroid Hormones | GDM Diagnosis (n=308) | | |
| --- | --- | --- | --- |
|  | OR | 95% CI | *P* |
| TT (ng/dL) | 2.90 | 1.08, 7.83 | 0.04 |
| fT (ng/dL) | 3.11 | 1.17, 8.27 | 0.02 |
| E1 (pg/mL) | 2.77 | 1.10, 6.94 | 0.03 |
| E2 (pg/mL) | 2.24 | 0.68, 7.35 | 0.18 |
| E3 (pg/mL) | 0.93 | 0.53, 1.62 | 0.79 |
| TT/E2 | 1.53 | 0.64, 3.67 | 0.34 |

Note. Maternal age, race/ethnicity, parity, gestational age of blood draw, early-pregnancy BMI, and infant sex were adjusted in the primary models. GDM: gestational diabetes; TT: total testosterone; fT: free testosterone; E1: estrone; E2: estradiol; E3: estriol.

Supplementary Table 6. Associations of early-pregnancy BMI with first trimester sex steroid hormones.

| Sex Hormones | Model (n=299) | | |
| --- | --- | --- | --- |
|  | Coefficient | 95% CI | *P* |
| TT (ng/dL) | 0.005 | -0.005, 0.015 | 0.34 |
| fT (ng/dL) | 0.014 | 0.004, 0.025 | 0.006 |
| E1 (pg/mL) | -0.02 | -0.032, -0.009 | 0.001 |
| E2 (pg/mL) | -0.014 | -0.022, -0.006 | <0.001 |
| E3 (pg/mL) | -0.015 | -0.031, 0.001 | 0.07 |
| TT/E2 | 0.019 | 0.009, 0.029 | <0.001 |

Note. Log transformed sex steroid hormones as the outcomes, respectively. Maternal age, race/ethnicity, parity, gestational age of blood draw, infant sex, and fertility treatment were adjusted in the models. BMI: body mass index; TT: total testosterone; fT: free testosterone; E1: estrone; E2: estradiol; E3: estriol.

Supplementary Table 7. Associations of log-transformed first trimester sex steroid hormones and continuous glucose levels and GDM diagnosis in mid-late pregnancy without adjustment for early-pregnancy BMI.

| Sex Steroid Hormone | Glucose Levels (n=284) | | | GDM Diagnosis (n=308) | | |
| --- | --- | --- | --- | --- | --- | --- |
|  | Coefficient | 95% CI | *P* | OR | 95% CI | *P* |
| TT (ng/dL) | 5.54 | 0.26, 10.81 | 0.04 | 3.97 | 1.63, 9.69 | 0.002 |
| fT (ng/dL) | 6.84 | 1.86, 11.83 | 0.007 | 4.11 | 1.76, 9.62 | 0.001 |
| E1 (pg/mL) | 2.90 | -1.61, 7.40 | 0.21 | 2.95 | 1.31, 6.64 | 0.01 |
| E2 (pg/mL) | 3.42 | -3.17, 10.01 | 0.31 | 2.25 | 0.80, 6.37 | 0.13 |
| E3 (pg/mL) | 2.39 | -0.78, 5.56 | 0.14 | 0.99 | 0.62, 1.58 | 0.98 |
| TT/E2 | 3.04 | -2.03, 8.11 | 0.24 | 1.87 | 0.91, 3.87 | 0.09 |

Note. Maternal age, race/ethnicity, parity, gestational age of blood draw, and infant sex were adjusted in all models. Fertility treatment was adjusted in the models with glucose levels as the outcome. GDM: gestational diabetes; BMI: body mass index; TT: total testosterone; fT: free testosterone; E1: estrone; E2: estradiol; E3: estriol.

Supplementary Table 8. Mixed Effects Models of the Associations of GDM Diagnosis with Sex Steroid Hormones in the 2^nd^ and 3^rd^ Trimesters

| Sex Steroid Hormones | Primary Model (n=284) | | | Model additionally adjusting for 1^st^ trimester SSH (n=281) | | |
| --- | --- | --- | --- | --- | --- | --- |
|  | Coefficient | 95% CI | *P* | Coefficient | 95% CI | *P* |
| TT (ng/dL) | 0.12 | -0.14, 0.39 | 0.35 | -0.19 | -0.36, -0.02 | 0.03 |
| fT (ng/dL) | 0.16 | -0.12, 0.44 | 0.27 | -0.10 | -0.31, 0.12 | 0.36 |
| E1 (pg/mL) | 0.29 | 0.02, 0.56 | 0.03 | 0.01 | -0.18, 0.19 | 0.95 |
| E2 (pg/mL) | 0.14 | -0.02, 0.29 | 0.08 | 0.06 | -0.06, 0.17 | 0.35 |
| E3 (pg/mL) | 0.08 | -0.07, 0.23 | 0.27 | 0.08 | -0.07, 0.23 | 0.29 |
| TT/E2 | -0.01 | -0.28, 0.26 | 0.94 | -0.14 | -0.34, 0.06 | 0.17 |

Note. Maternal age, race/ethnicity, parity, gestational age of blood draw, fertility treatment, early-pregnancy BMI, GWG by the end of 2^nd^ and 3^rd^ trimesters, and infant sex were adjusted in the primary models. GDM: gestational diabetes; TT: total testosterone; fT: free testosterone; E1: estrone; E2: estradiol; E3: estriol.

SSH

GWG

GDM

Supplementary Figure 1. Mediation model of sex steroid hormones, gestational weight gain, and gestational diabetes. SSH, sex steroid hormone; GWG, gestational weight gain; GDM, gestational diabetes.


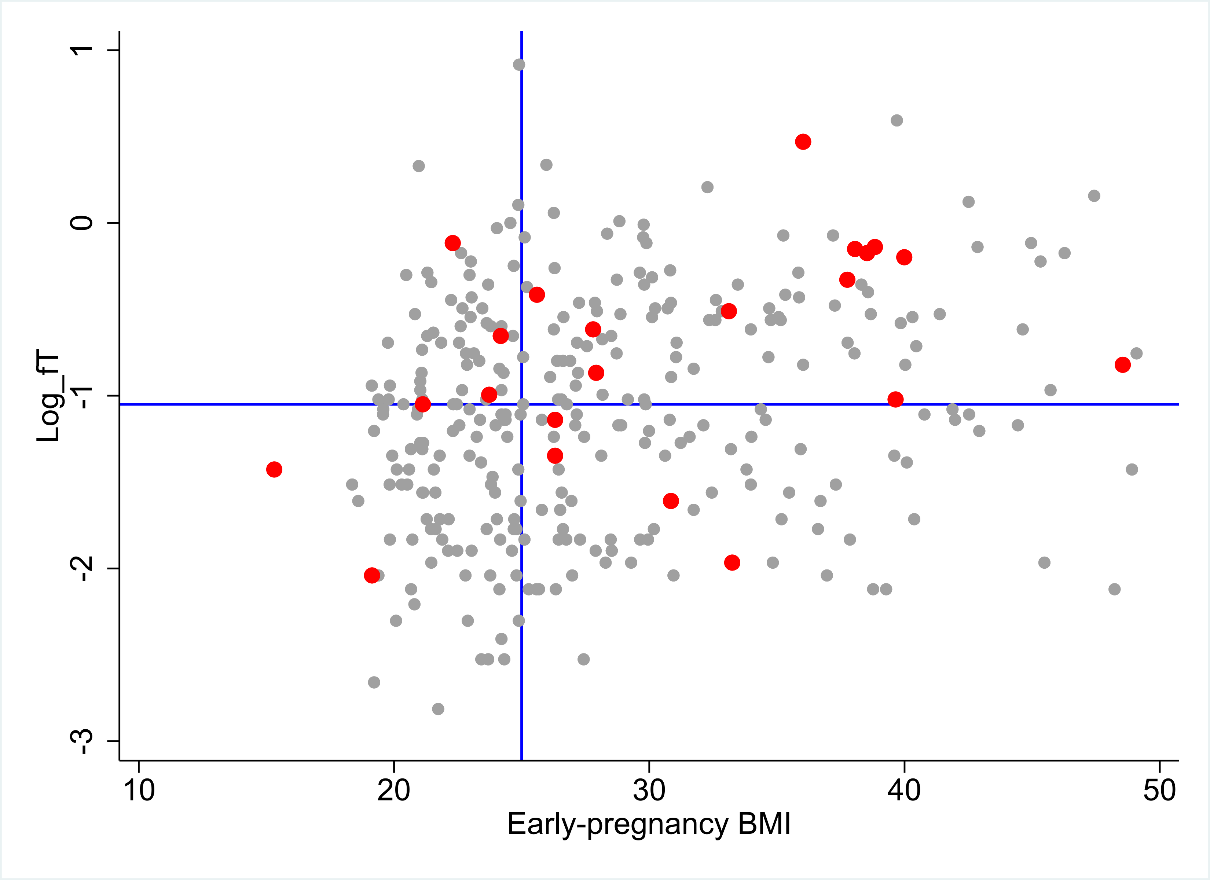


Supplementary Figure 2. The relationship among early-pregnancy free testosterone, early-pregnancy BMI and GDM diagnosis. Red dots indicate participants with GDM. The horizontal blue line indicates the median level of free testosterone. The vertical blue line indicates a BMI of 25 kg/m^2^.
