# Supplementary material for: Association between sex steroid hormones and subsequent hyperglycemia during pregnancy
Source: Front Endocrinol (Lausanne). 2023 Sep 8;14:1213402. doi: 10.3389/fendo.2023.1213402 (PMC10520461; doi:10.3389/fendo.2023.1213402)
Supplement: Supplementary file 2 [file Presentation_1.pdf]

Supplementary materials:

Screening of potential undiagnosed PCOS cases:

Two sets of questions were used to identify potential undiagnosed PCOS cases.

The first set of questions were used to assess menstrual periods:
(1) Menstrual irregularity: “Do you typically have fewer than eight periods per year when not taking hormonal contraceptives?” and “When you were not using birth controls pills/hormonal contraception, how regular were your menstrual periods in the past year?” Participants who answered “Yes” to the first question or “Very irregular” or “Didn’t usually have bleeding periods” to the second question were considered as positive for menstrual irregularity.

The second set of questions were used to assess PCOS related symptoms, including hirsutism and acne:
(2) Hirsutism/acne: “Before pregnancy, have you ever had excess hair growth on your upper lip, chin, neck, chest or abdomen?” and “Before pregnancy, have you ever had acne on your chin, neck, and/or chest?” Participants who answered “Yes” to the first question or “Yes, a lot” to the second question were considered as positive for hirsutism/acne

Participants who were positive for both sets of questions, were categorized as potential undiagnosed PCOS cases and excluded in sensitivity analyses.
